# Matrix-Assisted Laser Desorption Ionization Time of Flight (MALDI-TOF) as an Indispensable Tool in Diagnostic Bacteriology: A Comparative Analysis With Conventional Technique

**DOI:** 10.7759/cureus.36984

**Published:** 2023-03-31

**Authors:** Akanksha Gupta, Jyotsna Agarwal, Vikramjeet Singh, Anupam Das, Manodeep Sen

**Affiliations:** 1 Microbiology, Dr. Ram Manohar Lohia Institute of Medical Sciences, Lucknow, IND

**Keywords:** rapid diagnosis, rare microorganisms, diagnostic stewardship, conventional identification, biochemical analysis

## Abstract

Introduction: Owing to its accurate diagnosis, rapid turnaround time, cost effectivity, and less rates of error, Matrix-assisted Laser Desorption Ionization Time of Flight (MALDI-TOF) has replaced most of the phenotypic methods of identification. Thus, the objective of this study was to compare and evaluate MALDI-TOF MS to conventional biochemical-to identify bacterial microorganisms.

Methods: Different bacterial species isolated from 2010 to 2018 (pre-MALDI-TOF era), using routine bio-chemicals were compared to bacterial species isolated from 2019 to August 2021 (post MALDI-TOF), using MALDI-TOF, in the microbiology laboratory of a tertiary care hospital in North India. Chi-Square test (χ2) was used for the evaluation of bacterial identification between biochemical tests and MALDI-TOF MS association with a 95% confidence interval, considering wrong identification in genera or at a species level.

Results: Many different and new genera and species of bacteria could be identified using MALDI-TOF, which was not possible using only routine manual bio-chemicals like *Kocuria rhizophilus*, *Rothia mucilaginosa,* *Enterococcus casseliflavus, Enterococcus gallinarum, Leuconostoc, Leclercia adecarboxylata, Raoultella ornithological, Cryseobacterium indologenes.*

Conclusion: Each of the newly identified bacteria played an important role in deciding treatment. Wide use of the MALDI-TOF system will not only strengthen diagnostic stewardship but also encourage antimicrobial stewardship programs.

## Introduction

Mass spectrometry (MS) has been utilized for several decades as a diagnostic tool [1]. In 1975, Anhalt and Fenselau demonstrated that different microorganisms especially focusing on bacteria and fungus show distinct spectra of protein mass, thus can be used for characterization and this method known as matrix-assisted laser desorption/ionization time-of-flight (MALDI-TOF) MS for characterization of bacteria. In the last 10 years, MALDI-TOF MS has become an important part of microbiology laboratories as a diagnostic tool, even replacing most other identification techniques like phenotypic biochemical methods and latex agglutination tests for fastidious organisms [2].

This identification method owing to its rapid turnaround time and high diagnostic accuracy has gained importance in very less time [3]. MALDI-TOF MS has even been used for the detection and identification of viruses, protozoans, and arthropods in research settings [4-6]. In MALDI-TOF MS there is neither requirement for highly skilled laboratory technicians for sample preparation procedures nor there is need for complex additional laboratory infrastructure. Inexpensive, less chances of contamination and rapid and prompt identification results are other advantages of MALDI-TOF MS. Earlier it was believed that MALDI-TOF requires colonies of a given pathogen on culture media, but it’s not the case today. Instead, studies have come which reported accurate identification of pathogens directly from blood culture broths flagged positive [6,7]. A recent study [8] has proposed a new platform for the analysis of mixed bacterial culture using MALDI-TOF spectra (instead of only a single pathogen). Hence, this procedure can be applied directly to other body fluids, urine, respiratory specimens, and fecal samples, thus further increasing its importance in clinical practice.

Diagnosis plays a central role in effective treatment but the requirements for its set up like a basic laboratory, light microscopes, and trained laboratory technicians might not be available in isolated areas of our country. Thus, this study was done to compare and evaluate the MALDI-TOF MS technique to traditional biochemical-base methods to identify bacterial microorganisms from growth on culture plates that were misidentified at the species level.

## Materials and methods

This is a retrospective observational study conducted in a tertiary care hospital in North India. In this study distribution of different microorganisms isolated from different specimens is compared before and after the introduction of MALDI-TOF (August 2019). Before the introduction of MALDI-TOF in our laboratory setup, the identification of organisms was mostly done by biochemical analysis, and sometimes by Vitek 2 system. After the introduction of MALDI-TOF, all isolates are identified using MALDI-TOF. For a brief period of time, Vitek 2 system was used for the identification of organisms of critical patients. But MALDI-TOF was found to have a better performance than Vitek 2 system in identifying bacteria. Moreover, MALDI-TOF MS is a quick and cost-effective technique with the potential to be used in place of conventional methods of identifying common bacterial isolates. Thus, MALDI-TOF was solely used for the identification of bacteria. So, the organisms isolated between the years 2010 and 2018 are compared with isolated ones identified from the year 2019 to August 2021, i.e., pre- and post-introduction of MALDI-TOF.

Routine manual biochemicals for identification of Gram-negative bacteria that were used in the pre-MALDI-TOF era are indole production, methyl red, citrate utilization, Triple Sugar Iron (TSI) agar, urease production, Pheny Pyruvic Acid (PPA), sugars (glucose, lactose, maltose, mannitol, sucrose, and sorbitol). Depending upon different isolates, other sugars were used accordingly. For suspected *Enterococci*, bile esculin and arabinose fermentation was used for speciation. *Streptococcus *spp was differentiated from *Enterococci *on the basis of growth on MacConkey media and hemolysis on blood agar. Further differentiated by optochin sensitivity, bacitracin sensitivity, bile solubility, CAMP test, and other tests as needed. For catalase-positive, Gram-positive cocci, Coagulase test, DNAse agar, and carbohydrate oxidation-fermentation tests were routinely used. MALDI-TOF MS was performed as per the manufacturer’s guidelines. A chi-square test (χ2) with a 95% confidence interval was applied for the evaluation of the association between biochemical tests and MALDI-TOF MS for bacterial identification.

Ethics

The present study related to MALDI-TOF MS identification was approved by the Institute Ethical Committee (IEC 48/20, dated April 20, 2020).

## Results

Catalase positive Gram-positive cocci

All the catalase-positive Gram-positive cocci isolated from various samples used to be treated as either *Staphylococcus *spp or *Micrococcus *spp. The above two were differentiated and *Staphylococcus *was speciated manually using the above-mentioned tests.

**Figure 1:**
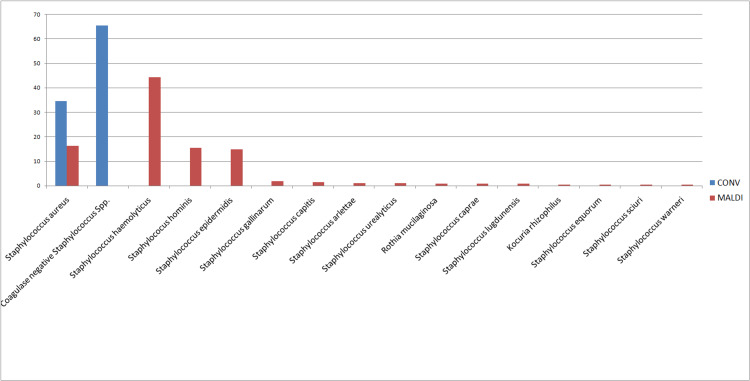
Comparison between Percentage distribution of various catalase positive Gram-positive cocci in pre (CONV) and post (MALDI) MALDI-TOF analysis

Now using MALDI-TOF, not only could we speciate all the CONS (Coagulase Negative *Staphylococcus *spp.), but we could also identify other catalase-positive Gram-positive cocci, such as *Kocuria rhizophilus* (0.4%) and *Rothia mucilaginosa* (0.74%). The various *Staphylococcus *species identified by MALDI-TOF are in Figure 1.

Catalase negative Gram-positive cocci

**Figure 2:**
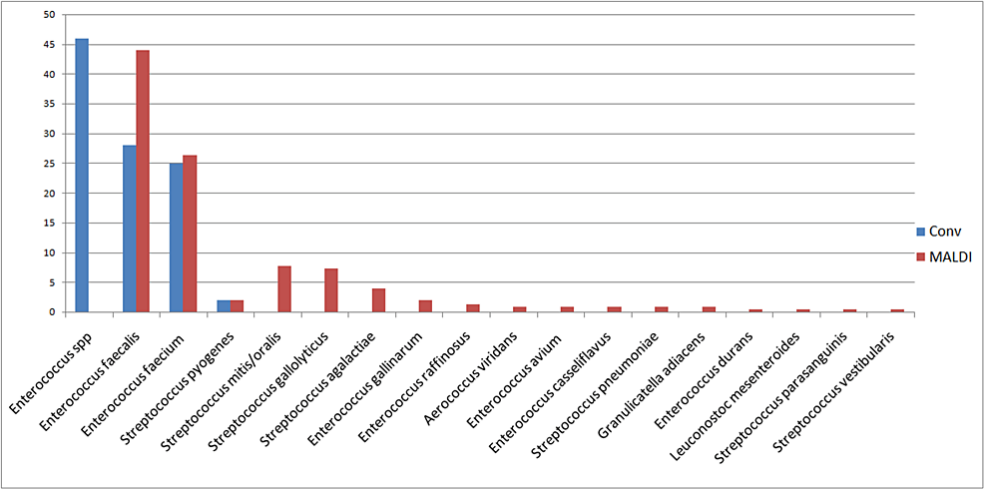
Percentage distribution of various catalase negative Gram-positive cocci in pre (CONV) and post (MALDI) MALDI-TOF analysis

All the catalase negative Gram-positive cocci in pairs or chains were considered to be either *Enterococcus *or *Streptococcus*. Enterococci were further differentiated into *E. faecalis* or *E. faec*ium on the basis of arabinose fermentation. So, the isolates identified during the pre MALDI era were *E. faecalis*, *E. faecium*, *Enterococcus *spp, Viridans group of *Streptococci *and *Streptococcus pyogenes*. The number of *Enterococcus *and *Streptococcus *species that could be identified increased when MALDI was used for identification. The various species isolated are in Figure 2.

Lactose fermenters Gram-negative bacilli

All the Gram-negative bacilli which were lactose fermenters, oxidase negative and catalase positive were considered to be of the order Enterobacterales. These were further classified manually on the basis of routine bio-chemicals mentioned above. By this method, we could have misidentified few species or could have missed the rare species of Enterobacterales order.

**Figure 3:**
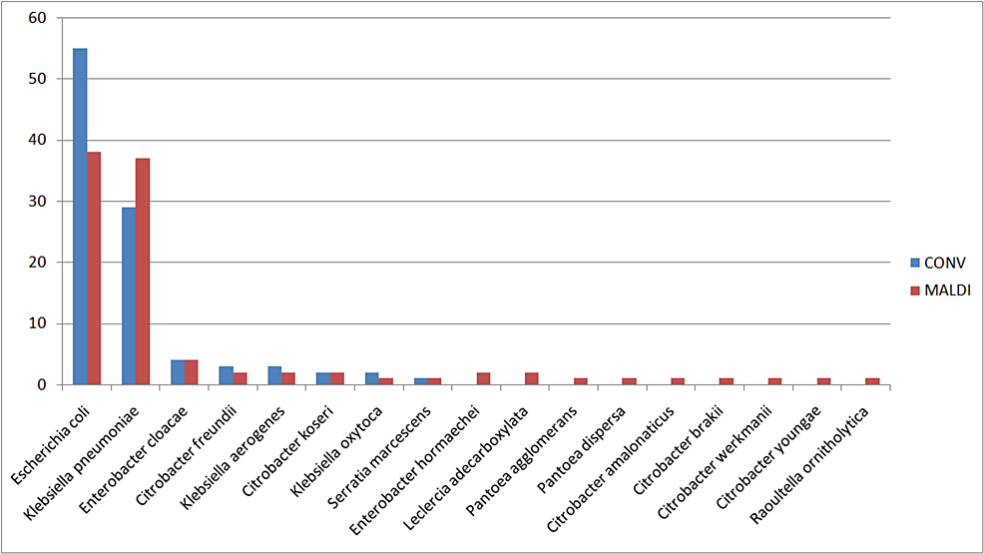
Percentage distribution of various lactose fermenting Gram-negative bacilli in pre (conv) and post-MALDI-TOF analysis

Since MALDI-TOF is based on the identification of ribosomal proteins, it could also identify the few less commonly isolated species with clear distinction from related species. The various organisms isolated from manual and MALDI-TOF analyses are given in Figure 3.

Oxidase negative non-lactose fermenters

The isolates appearing as non-lactose fermenters (NLF) on Mac Conkey were tested for oxidase test. Oxidase negative NLFs were further differentiated into genera and species by various biochemicals mentioned above. Using these biochemicals, we could identify common species of *Acinetobacter*, *Proteus*, *Providencia,* and *Morganella*. In the post MALDI-TOF era, apart from the above-mentioned genus, we could also identify other isolates like *Stenotrophomonas*, *Serratia, *etc. (Table I).

**Table 1:** Percentage distribution of various lactose non fermenting, oxidase negative Gram-negative bacilli in pre (Conventional) and post-MALDI-TOF analysis

Pre (Conventional) Analysis	Post MALDI-TOF analysis
Acinetobacter baumannii (44.7%)	Acinetobacter baumannii (48.4%)
Acinetobacter lwoffii (15%)	Stenotrophomonas maltophilia (13.2%)
Proteus mirabilis (13.7%)	Morganella morgannii (7.6%)
Acinetobacter species (10.7%)	Serratia marcescens (7.6%)
Providencia rettgeri (4.4%)	Proteus mirabilis (7.4%)
Morganella morgannii (3.9%)	Providencia rettgeri (3.6%)
Proteus species (3%)	Providencia stuartii (3.3%)
Proteus vulgaris (2.5%)	Acinetobacter junii (1.6%)
Providencia stuartii (1.5%)	Acinetobacter johnsonii (1.3%)
Providencia species (0.5%)	Salmonella enterica (0.9%)
Salmonella species (0.1%)	Acinetobacter calcoaceticus (0.7%)
	Acinetobacter pittii (0.7%)
	Acinetobacter schindleri (0.7%)
	Proteus vulgaris (0.7%)
	Acinetobacter lwoffii (0.8%)
	Acinetobacter haemolyticus (0.4%)
	Acinetobacter radioresisten (0.2%)
	Acinetobacter ursingii (0.2%)
	Moraxella osloensis (0.4%)
	Providencia penneri (0.4%)

Oxidase positive Gram-negative bacteria

Before the introduction of MALDI-TOF in our set up, all oxidase positive non lactose fermenting Gram negative bacteria were considered to be *Pseudomonas*. *Pseudomonas* was speciated only as *Pseudomonas* aeruginosa on the basis of green pigment, rest all were reported as *Pseudomonas* species.

**Figure 4:**
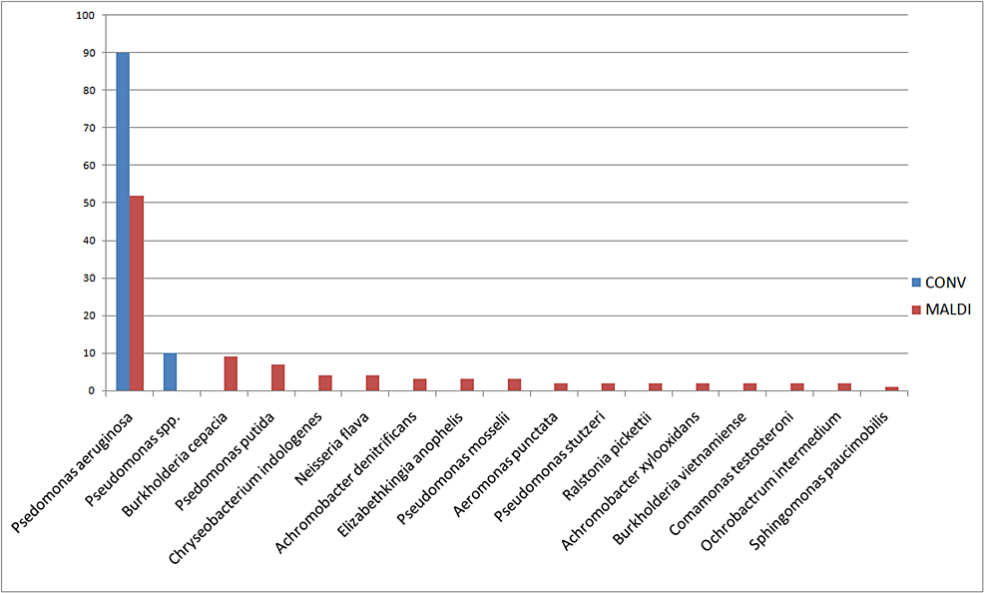
Percentage distribution of various lactose non fermenting oxidase positive Gram-negative bacilli in pre (conv) and post (MALDI) MALDI-TOF analysis

After the introduction of MALDI-TOF not only could we identify many species of *Pseudomonas*, but even other not so uncommon oxidase positive Gram-negative genus. The distribution of the various oxidase positive Gram-negative bacteria isolated in pre- and post-MALDI-TOF era is given in Figure 4.

## Discussion

For decades, conventional identification and classification of bacteria have been carried out on the basis of biochemical, metabolic, and antigenic properties. However, presently microorganisms are being detected primarily on the basis of mass-to-charge ratio and protein analysis. Especially, when there is an actual need for rapid identification of newly emerging microorganisms and their ever-evolving resistance trend, it’s high time that we should not focus only on antimicrobial stewardship but also on diagnostic stewardship. The present study was focused on how MALDI-TOF MS has helped us in identifying rare and exotic microorganisms of different genera and species which were earlier with conventional biochemical usually considered as commonly found microorganisms like Coagulase-negative *Staphylococcus *spp, *Pseudomonas *spp, *Enterococcus *spp, etc. After the involvement of MALDI TOF MS, we have significantly identified many microorganisms and are able to report those microorganisms and their intrinsic resistance pattern for better provision of treatment to patients.

Catalase positive Gram-positive cocci

From 2015 to 2017, we reported most Gram-positive, catalase-producing cocci into either *Staphylococcus aureus* (35%) or CoNS (65%). Eleven of the 30 CoNS species are known to be commensals in humans but may also have strong pathogenic potential depending on the site of infection involved. Species such as *S. haemolyticus*, *S. warne*ri, *S. hominis*, *S. capitis*, *S. intermedius*, *S. schleiferi*, and* S. simulans* are not so common pathogens constitute 75% of CoNS detected at our center after the inclusion of MALDI-TOF MS in clinical microbiology diagnostic services. Speciating CoNS into various species helps in epidemiological studies. Moreover, it also helps the clinician in deciding whether the isolated species could be the causative organism or is just a normal commensal, so as not to miss out on treating the clinically important species and at the same time to prevent over-treating the otherwise clinically nonsignificant Staphylococcal species [9].

Clinicians might tend to ignore CoNS as the causative agent of bloodstream infections considering it as normal skin flora hence a contaminant. *S. lugdunesi*s is a newly recognized species in various infections and has been identified as one of the emerging CoNS associated with serious BSI, especially in patients with critical prosthetic materials (cardiac pacemakers, catheters), thus speciation of CoNS is necessary [10]. Similarly, two important members of the Micrococcaceae family, *R. mucilaginosa* and *K. rhizophilus* could otherwise be misdiagnosed as CoNS and ignored as normal commensal flora, while these two organisms have been implicated as causative organisms in various infections like UTI, cholecystitis, bacteremia, bloodstream infections, etc., especially in pediatric patients [11,12].

Different Staphylococcal species may show different antimicrobial susceptibility patterns. A study quoted high resistance of *S. haemolyticus* against penicillin and oxacillin, and similarly high resistance of *S. homini*s and *S. epidermid*is against erythromycin. *S. lugdunensi*s was seen to be susceptible to most of the commonly used antistaphylococcal drugs, while *S. haemolyticus* showed multidrug resistance against most of the commonly used antibiotics [13]. Thus, speciation of Staphylococcus into various species (not just CoNS) is required not only to know when to treat but also to have an idea about which drugs to be used to treat depending on the resistance pattern of different Staphylococcal species.

Catalase negative Gram-positive cocci

As shown in the result, before the MALDI-TOF era, Catalase negative Gram-positive cocci were only recognized as either, *Enterococcus faecalis*/*Enterococcus faecium* or *Enterococcus *species. It was only after MALDI-TOF introduction could we identify other not-so-infrequent species like *Enterococcus casseliflavus*, *Enterococcus gallinarum*, and *Leuconostoc*. Though not so commonly isolated from clinical specimens, these species are associated with serious invasive infections. Why the identification of the above species is so important because of their intrinsic resistance to vancomycin. In the absence of the correct identification, clinicians might start vancomycin as an empirical treatment with no clinical improvement [14,15]. MALDI-TOF helps in the identification of fastidious organisms like *Aerococcus viridans* and helps in forming a better correlation between patients’ symptoms and their treatment. *Aerococcus *morphologically resemble the viridans group of Streptococci, so may be ignored as normal flora if not correctly identified. A study highlights accurate and timely identification of *Aerococcus *species to start correcting antimicrobial therapy so as to prevent any serious complications like pyelonephritis, urosepsis, or even death. This further highlights the importance of correct identification of microorganisms which may not always be possible using only some routine bio-chemicals [16].

Lactose fermenters Gram-negative bacilli

It is important to speciate the different lactose fermenting Gram-negative bacilli, including the different members of the order Enterobacterales as different bacteria have intrinsic resistance against different antimicrobials. For example, *Klebsiella pneumoniae* is intrinsically resistant to ampicillin, while *Escherichia coli* has no intrinsic resistance.

Few species that cannot be confirmed by manual biochemicals were isolated and confirmed by MALDI-TOF. One such microorganism is *Serratia marcescens*. It accounts for around 1%-2% of nosocomial infections, mostly UTIs, respiratory tract infections, surgical tissues, and soft tissue infections. The main importance of correctly identifying this organism is its ability to produce beta-lactamase, hence many beta-lactams are ineffective. Further, this isolate has intrinsic resistance against ampicillin, amoxicillin-clavunate, ticarcillin and first-generation cephalosporins like cefazolin [17].

*Leclercia adecarboxylata* is a rare genus of the order Enterobacterales. It is rarely reported as a human pathogen, but there are case reports showing it to be an emerging human pathogen. It is phenotypically similar to *E. coli* and even automated systems too fail to differentiate between the two. The absence of documented case reports may be due to the lack of correct identification of this organism. This organism was isolated in this study in a bloodstream infection case; this was made possible due to MALDI-TOF [18].

Another not-so-common isolate identified was *Raoultella ornitholytica*. It is also a member of the Enterobacterales order and is considered to be an opportunistic infection in children (mostly neonates and less than three years of age) and adults (hospital-acquired infections following invasive procedures). It is important to prevent its spread as it can lead to severe sepsis and multi-organ failure. Also, this organism is associated with high rates of antimicrobial resistance [19].

Oxidase-negative non-lactose fermenters

*Stenotrophomonas maltophilia* is another new emerging multi-drug resistant organism mostly associated with respiratory tract infections. It can spread in ICU settings by direct contact and through contaminated hands of the health care workers. It is thus important to monitor its persistence and spread within the community. This isolate is associated with intrinsic resistance against quite a number of antimicrobials, including carbapenems, aminoglycosides, most of the third-generation cephalosporins, and other β-lactams, hence treatment will depend on its correct identification. It may be misidentified as *Burholderia cepaci*a [20].

Oxidase-positive Gram-negative bacteria

MALDI-TOF analysis led to the identification of many new bacterial species as compared to previous manual biochemical analyses. Most of the *B. cepacia* were wrongly identified as *Pseudomonas *species. The treatment options of the two differ considerably, for example, Cotrimoxazole along with other antibiotics is the drug of choice for the former, while no role of cotrimoxazole in *Pseudomonas *infections [21].

Similarly, excessive use of colistin and tigecycline therapy has led to collateral damage and increased *Cryseobacterium indologenes* infection in healthcare settings. This bacterium too had been misidentified as *Pseudomonas *species in the absence of MALDI-TOF, which would have led to increased treatment with colistin therapy. Selecting correct antimicrobials is difficult due to the lack of proper guidelines for their treatment [22].

Limitations of MALDI-TOF

In spite of the above advantages of MALDI-TOF in the identification of microorganisms, hence early and appropriate treatment, there are a few limitations of MALDI-TOF. One of the important limitations is its variability in correctly identifying the capsulated microorganisms like Pneumococci, Viridans group of *Streptococci*, *K. pneumoniae*, and *Haemophilus influenzae*. Furthermore, MALDI-TOF cannot differentiate between *E. coli* and *Shigella dysenteriae*. The six closely related species of *Enterobacter cloacae* complex (*E. asburiae, E. cloacae*,* E. hormaechei*,* E. kobei*,* E. ludwigii*,and *E. nimipressuralis*) cannot be differentiated.

## Conclusions

Even though the correct identification up to the species level may not always drastically change the empirical therapy but it is still important to know the isolation up to the species level. Firstly, because it will help in epidemiological studies and to know the different species being isolated. Secondly, there are some species that are known to have an inherent resistance to some of the commonly used drugs against that genus. Using such an inherently resistant drug will not be of any use to the patient, thus knowing the isolates to the species level will help in preventing clinicians from giving such a drug. It is only with the correct identification of the organisms that we can get to know the various microorganisms which were routinely considered to be saprophytes are now being isolated as causative species.

The commencement of MALDI-TOF MS into clinical microbiology has made precise and speedy identification of microorganisms feasible, thus improving the diagnosis and reducing the time to more appropriate empirical therapy. As these diagnostic facilities continue to improve and become more widely available, the practice of diagnostic stewardship will be encouraged and transformed further along with antimicrobial stewardship programs.
